# Simultaneous Measurement of Benzo[*a*]pyrene-induced *Pig-a* and *lacZ* Mutations, Micronuclei and DNA Adducts in Muta™ Mouse

**DOI:** 10.1002/em.20688

**Published:** 2011-10-04

**Authors:** Christine L Lemieux, George R Douglas, John Gingerich, Souk Phonethepswath, Dorothea K Torous, Stephen D Dertinger, David H Phillips, Volker M Arlt, Paul A White

**Affiliations:** 1 Mechanistic Studies Division, Environmental and Radiation Health Sciences DirectorateHECSB, Health Canada, Ottawa, ON, Canada; 2 Litron LaboratoriesRochester, New York; 3 Section of Molecular Carcinogenesis, Institute of Cancer ResearchSutton, Surrey, United Kingdom

**Keywords:** phosphatidylinositol glycan complementation group A, genotoxicity, reduction, mutagenic efficiency, dose response kinetics

## Abstract

In this study we compared the response of the *Pig-a* gene mutation assay to that of the *lacZ* transgenic rodent mutation assay, and demonstrated that multiple endpoints can be measured in a 28-day repeat dose study. Muta™Mouse were dosed daily for 28 days with benzo[*a*]pyrene (BaP; 0, 25, 50 and 75 mg/kg body weight/day) by oral gavage. Micronucleus (MN) frequency was determined in reticulocytes (RETs) 48 hr following the last dose. 72 h following the last dose, mice were euthanized, and tissues (glandular stomach, small intestine, bone marrow and liver) were collected for *lacZ* mutation and DNA adduct analysis, and blood was evaluated for *Pig-a* mutants. BaP-derived DNA adducts were detected in all tissues examined and significant dose-dependent increases in mutant *Pig-a* phenotypes (i.e., RET^CD24-^ and RBC ^CD24-^) and *lacZ* mutants were observed. We estimate that mutagenic efficiency (i.e., rate of conversion of adducts into mutations) was much lower for *Pig-a* compared to *lacZ*, and speculate that this difference is likely explained by differences in repair capacity between the gene targets, and differences in the cell populations sampled for *Pig-a* versus *lacZ*. The BaP doubling doses for both gene targets, however, were comparable, suggesting that similar mechanisms are involved in the accumulation of gene mutations. Significant dose-related increases in % MN were also observed; however, the doubling dose was considerably higher for this endpoint. The similarity in dose response kinetics of *Pig-a* and *lacZ* provides further evidence for the mutational origin of glycosylphosphatidylinositol (GPI)-anchor deficiencies detected in the *Pig-a* assay. Environ. Mol. Mutagen. 2011. © 2011 Wiley-Liss, Inc.

## INTRODUCTION

Data for the induction of gene mutations in vivo form an increasingly important component of hazard and risk assessment. One widely used and well-validated system for assessment of gene mutations in vivo is the transgenic rodent (TGR) mutation assay system (for review see [Lambert et al.,^2005^; OECD,^2009^]). Commonly used versions of the TGR assays include the *lacZ* bacteriophage mouse or Muta™Mouse [Gossen et al.,^1989^; Vijg and Douglas^1996^], the *lacI* or BigBlue™ rodents (rat and mouse), the *lacZ* plasmid mouse, and the gpt-delta mouse [Lambert et al.,^2005^]. The regulatory importance of these assays has been recognized by the Organization for Economic Cooperation and Development (OECD) and an OECD Test Guideline (no. 488) was approved in 2011 [OECD,^2011^].

Another promising assay for in vivo gene mutation testing is the recently developed *Pig-a* mutation assay. The *Pig-a* assay has multispecies capability and, when fully validated, may constitute an effective tool for in vivo mutation research and hazard assessment [Bryce et al.,^2008^; Phonethepswath et al.,^2008^; Dobrovolsky et al.,^2010^]. The *Pig-a* assay is based on detection of glycosylphosphatidylinositol (GPI) anchored proteins on the cell surface of circulating blood cells (reticulocytes or RETs) and red blood cells (RBCs). The *Pig-a* (phosphatidylinositol glycan complementation group A) gene product is involved in the first step of GPI anchor biosynthesis, and since it is the only X-linked gene involved in the GPI anchor synthesis pathway, it is generally accepted that a single mutation at the *Pig-a* locus can prevent the anchoring of GPI anchored proteins (i.e., CD24 in mouse, or CD59 in rat). Mutant RBCs or RETs therefore lack cell surface expression of these proteins (RBC^CD24−^ or RET^CD24−^), and this phenotype can readily be detected by flow cytometry. For a detailed description of the assay, the reader is referred to [Phonethepswath et al.,^2008^]. Several research groups have been working towards the validation of this assay (for example see [Miura et al.,^2008^; Phonethepswath et al.,^2010^; Kimoto et al.,^2011^]) and it is the focus of this Special Issue; however, much work still remains to be completed before the assay can be routinely used for regulatory genetic toxicity testing.

This study contributes to the validation of the *Pig-a* endpoint by comparing its responsiveness to that of the well established *lacZ* TGR mutation assay for the well-known mutagenic carcinogen benzo[*a*]pyrene (BaP). The results of this study provide useful information on how induction of mutant *Pig-a* phenotypes compares to that of a well established and validated mutation target, (i.e., *lacZ*), in the same animals.

An important feature of this study is the integration of multiple genotoxicity endpoints in a single experiment. This facilitates reduction of the number of animals used for testing, as recommended by the European Centre for the Validation of Alternative Methods (ECVAM) [Pfuhler et al.,^2009^]. Although there has been progress in the integration of multiple endpoints into 28-day repeat dose studies (for example, see [Dertinger et al.,^2010^; Shutsky et al.,^2010^]), further work is required in order to determine the most appropriate endpoints, dosing regimes, and sampling times.

This study has three overall objectives: (1) to contribute to the validation of the *Pig-a* assay by comparing the response of the *Pig-a* assay to the well established *lacZ* mutation assay; (2) to assess how simultaneous measurement of multiple endpoints (for mutation, chromosome damage and DNA adducts) can be achieved in a single 28-day subchronic mouse study; and (3) to determine the utility of DNA adduct data in assessing the efficiency of mutation induction. We hypothesized that, although the two mutation assays examine different loci (i.e., *Pig-a* and *lacZ*), their responses would be similar given their presumed similar mechanisms of action. Conversely, we expected that the dose-response kinetics of BaP-induced micronucleus formation would be different, given that this cytogenetic damage endpoint is sensitive to different types of lesions.

## MATERIALS AND METHODS

### Chemicals

All chemicals were obtained from Sigma Aldrich Canada (Oakville, ON), except Tris-HCl, phosphate buffered saline (PBS), and proteinase-K, which were obtained from Invitrogen Canada (Burlington, ON).

### Animal Treatment

Twenty-five-week old male Muta™Mouse specimens (i.e., transgenic mouse strain 40.6) were dosed daily via oral gavage for 28 days with BaP dissolved in olive oil (25, 50, and 75 mg/kg body weight/day). Each dose group, including vehicle control, contained five animals. Following OECD Test Guideline 474 [OECD,^1997^], peripheral blood for MN analysis was collected from the saphenous vein 48 hr after the last treatment. Following a three-day sampling period after the final treatment, mice were anaesthetized with isofluorane to permit blood collection via cardiac puncture. This was immediately followed by cervical dislocation. 60 μl aliquots of the collected blood were processed for the *Pig-a* mutation endpoint analysis. Tissues, including liver, bone marrow, small intestine, and glandular stomach, were isolated, flash-frozen in liquid nitrogen, and stored at −80°C until use. One mouse, dosed i.p. with ethylnitrosourea (45 mg/kg body weight) two weeks prior to necrospsy was used as a positive control for the *Pig-a* assay. Mice were maintained under conditions approved by the Health Canada Animal Care Committee. Food and water were available *ad libitum* for the duration of the experiment.

### Genomic DNA Isolation

#### Glandular Stomach

Mucosal cells from glandular stomach were isolated and lysed according to [Brault et al.,^1999^]. Briefly, glandular stomach was thawed, and stomach mucosal cells were removed from the inner lining of the glandular stomach, and were homogenized in 5 ml lysis buffer (1 mM Na_2_EDTA, 100 mM NaCl, 20 mM Tris-HCl, pH 7.4), supplemented with 1% SDS (w/v) and 0.1 mg/ml Rnase A and incubated for 1 hr at 37°C. Proteinsase K (1 mg/ml) was added and cells were incubated at 37°C overnight with gentle shaking. Genomic DNA was isolated the day following lysis, using the phenol/chloroform extraction procedure described previously [Douglas et al.,^1994^; Vijg and Douglas^1996^]. Isolated DNA was dissolved in 100 μl TE buffer (10 mM Tris pH 7.6, 1 mM EDTA) and stored at 4°C until used.

#### Bone marrow

To collect bone marrow, femurs were flushed with PBS, the solution was briefly centrifuged, and the pellet was stored at –80°C. DNA was extracted as described above.

#### Small intestine

Epithelial cells were isolated from the jejunum of the small intestine using a method modified from [Tao et al.,^1993^] and [Trentin et al.,^1998^]. Frozen tissue was defrosted on ice and slit open in 1.5 ml cold “snapping buffer” (75 mM KCl, 20 mM EDTA). The tissue was passed quickly into and out of a 1-ml syringe (i.e., “snapped”) three times, and the buffer was discarded. The tissue was then snapped six to nine times in an additional 3 ml of buffer before the cell suspension was collected and centrifuged for 10 min at 1,500 g and 4°C. DNA was extracted from the cell pellet as described above.

#### Liver

Liver tissue was thawed and homogenized on ice using a motor-driven conical tissue homogenizer in 7 ml TMST buffer (50 mM Tris pH 7.6, 3 mM magnesium acetate, 250 mM sucrose, 0.2% (v/v) Triton X-100). The liver homogenate was centrifuged for 6 min at 800 g (4°C), the supernatant was discarded, and the pellet was washed twice more with TMST buffer as before. The pellet was suspended in 5 ml lysis buffer (10 mM Tris pH 7.6, 10 mM EDTA, 150 mM NaCl, 1% (w/v) SDS and 1mg/ml proteinase K (≥20 Units/mg). This suspension was incubated overnight at 37°C. Again, DNA was extracted as described above.

### DNA Adduct Analysis

DNA adducts were measured in bone marrow, liver, glandular stomach, and small intestine using the ^32^P-postlabeling method with nuclease P1 digestion enrichment [Phillips and Arlt,^2007^]. All enzymes and chemicals used for this assay were purchased from sources previously described in [Phillips and Arlt,^2007^]. Briefly, DNA samples (4 μg) were digested with micrococcal nuclease (120 mUnits) and calf spleen phosphodiesterase (40 mUnits), enriched and labeled as reported elsewhere [Philips and Arlt,^2007^]. Chromatographic conditions for thin-layer chromatography (TLC) on polyethyleneimine-cellulose (PEI-cellulose) plates (Macherey-Nagel, Düren, Germany) were [Arlt et al.,^2008^]: D1, 1.0 M sodium phosphate, pH 6; D3, 4 M lithium-formate, 7 M urea, pH 3.5; D4, 0.8 M LiCl, 0.5 M Tris, 8.5 M urea, pH 8. After chromatography, TLC sheets were scanned using a Packard Instant Imager (Dowers Grove, IL) and DNA adduct levels (RAL, relative adduct labeling) were calculated from adduct cpm, the specific activity of [γ-^32^P]ATP and the amount of DNA (pmol of DNA-P) used. An external BaP-diol-epoxide-DNA standard was used for identification of BaP-DNA adducts [Phillips and Castegnaro^1999^]. Results are expressed as DNA adducts/10^8^ nucleotides.

### Evaluation of Mutant Pig-a Phenotypes

RBCs were enriched by centrifugation with Lympholyte® and then washed with PBS. These coded specimens were shipped overnight on ice packs to Litron Laboratories for flow cytometric analysis. Enriched RBCs were incubated with anti-mouse-CD24-PE, washed, and then incubated with the cell permeable nucleic acid dye SYTO 13 as described in Phonethepswath et al. [2008]. For each specimen, the frequency of RETs and mutant phenotype RBCs was measured by interrogating approximately 10^6^ RBCs. The frequency of mutant phenotype RETs was measured by interrogating at least 3 × 10^5^ RETs per specimen using flow cytometry.

### lacZ Mutation Evaluation

The frequency of *lacZ* transgene mutants in genomic DNA isolated from liver, bone marrow, glandular stomach and small intestine was measured using the P-Gal positive selection assay as previously described [Vijg and Douglas,^1996^; Lambert et al.,^2005^]. λgt10*lacZ* DNA was rescued from genomic DNA using the Transpack™ lambda packaging system (Agilent, Mississauga, ON). Packaged phage particles were then mixed with the host bacterium (*Escherichia coli lacZ^−^*, *galE^−^*, *recA^−^*, pAA119 with *galT* and *galK*) [Gossen et al.,^1992^], plated on minimal medium containing 0.3% (w/v) P-Gal and incubated overnight at 37°C. Total plaque-forming units (pfu) were measured on concurrent titre plates that did not contain P-Gal. Mutant frequency (MF) is expressed as the ratio of mutant pfu to total pfu.

### Micronucleus Evaluation

For evaluation of micronuclei, blood samples (∼100 μl) were collected from the saphenous vein 48 hr following the last treatment [OECD,^1997^], and fixed according to MicroFlow® kit instructions. Coded specimens were shipped to Litron Laboratories for analysis, where they were stained and analyzed using a 3-color labeling method described by Dertinger et al. [2004]. Instrument settings were optimized with a MicroFlow-kit supplied biological standard (i.e., malaria-infected erythrocytes; Tometsko et al.,^1993^). The frequencies (%) of RETs, micronucleated RETs (MN-RET) and micronucleated NCEs (MN-NCE) were determined upon the acquisition of ∼20,000 RETs per specimen.

### Statistical Analyses

All data (i.e., adduct level, *lacZ* mutant frequency, mutant *Pig-a* phenotype frequency, and micronucleus frequency data) were analyzed by Poisson Regression using SAS v.9.1 (SAS Institute, Cary, NC). The data were fit to the model log(E(Y_*i*_)) = log *t*_*i*_ + ί **x**_*i*_, where E(Y_*i*_) is the expected value for the *i*th observation, ί is the vector of regressions coefficients, **x**_*i*_ is a vector of covariates for the *i*th observation, and *t*_*i*_ is the offset variable used to account for differences in observation count period (i.e., total pfu or total RETs). The offset (i.e., natural log) was given a constant coefficient of 1.0 for each observation, and log-linear relationships between mutant count and test article concentration were specified by a natural log link function. Type 1, or sequential analysis, was employed to examine the statistical significance of the BaP treatment, and custom contrasts were employed to evaluate the statistical significance of responses at each dose tested.

BaP doubling dose (i.e., the dose of BaP required to elicit a doubling in the spontaneous frequency) was calculated for each of the endpoints according to the method of Dubrova [2005], Eq. (1).


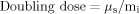
(1)

where μ_*s*_ is the spontaneous frequency, and *m*_*i*_ is the slope of the dose-response function.

The slopes of the dose-response functions, or *m*_*i*_, were calculated using ordinary least squares regression. Standard deviation for each doubling dose was calculated according to Sankaranarayanan and Chakraborty [2000]. The two-tailed Student's *t* test (*p* < 0.05), with the appropriate multiple test correction (i.e., Bonferroni correction), was used to compare doubling doses across endpoints.

## RESULTS

Transgenic mice were exposed to BaP daily for 28 days via oral gavage, and simultaneous measurements of DNA adduct frequency, micronucleus frequency, *lacZ* mutant frequency, and the frequency of mutant *Pig-a* phenotypes were carried out in an effort to better understand the responsiveness of *Pig-a* phenotype frequency relative to other established genotoxicity endpoints for the prototypical polycyclic aromatic hydrocarbon BaP. No overt signs of toxicity were observed in any dose group, including controls, and no statistically significant reductions in body weight were observed.

### DNA Adducts

**Fig. 1:**
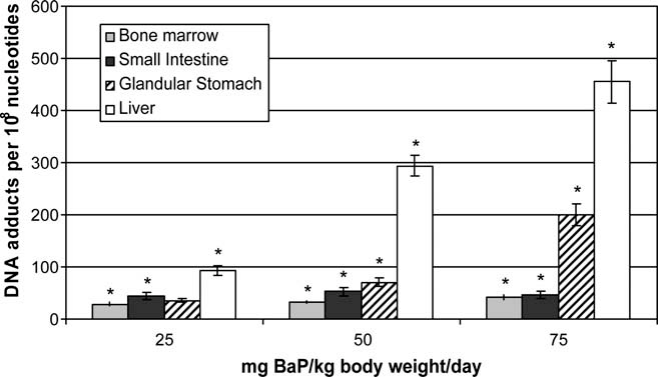
Relative adduct labeling (DNA adducts per 10^8^ nucleotides) of BaP-7,8-diol-9,10-epoxide-*N*^2^-deoxyguanosine (BPDE) adducts in tissues of BaP-treated Muta™ Mouse. Values are means of measurements from five separate animals (each DNA sample analysed in two independent postlabeling assays), and error bars represent standard error of the mean. (* indicates *p* < 0.05 compared to control).

DNA adducts were evaluated three days following the last treatment, via ^32^P postlabeling analysis, as a measure of internal dose of the active BaP metabolite to target tissues. Using an external BaP-diol-epoxide-DNA standard, the main adduct detected was identified as dG-*N*^2^-BPDE (BaP-7,8-diol-9,10-epoxide-*N*^2^-deoxyguanosine). DNA adducts were found to be present in all four of the tissues examined (Fig. 1), and all doses yielded DNA adduct levels that were significantly elevated compared to control (*p* < 0.05) in all tissues, with the exception of the low dose group (25 mg/kg body weight/day) in glandular stomach. Furthermore, DNA adduct formation was dose-dependent (dose effect *p* < 0.005). No DNA adducts were observed in the control animals.

### Mutagenic Response to BaP

#### Evaluation of Mutant Pig-a Phenotypes

**Fig. 2:**
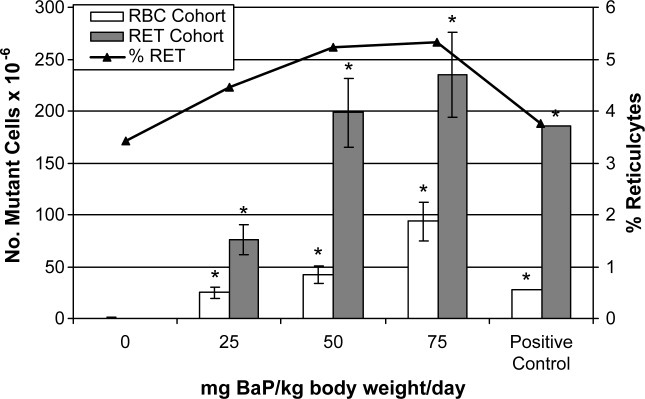
Frequency of mutant *Pig-a* phenotypes observed in reticulocytes (RETs) and red blood cells (RBCs). Bars represent the mean of measurement from five separate animals and error bars represent the standard error of the mean. The positive control represents the response observed for one mouse treated with 45 mg/kg body weight ethylnitrosourea (i.p.) two weeks prior to necropsy. (* indicates *p* < 0.05 compared to control) Note that no mutant phenotype RETs were observed in control animals, while in RBC, 0.40 × 10^−6^ ± 0.25 × 10^−6^ mutant phenotypes were observed.

The frequency of mutant *Pig-a* phenotypes was measured using flow cytometry for RBCs and RETs in blood samples obtained at necropsy (i.e., 72 hr following the last dose). Figure 2 summarizes the mutagenic response observed for the *Pig-a* locus. Dose-dependent increases in mutant phenotype RETs and RBCs (i.e., RET^CD24−^ and RBC^CD24−^) were observed in both cell populations (for RET^CD24−^ Poisson regression chi-square for test article concentration effect = 129.10, *p* < 0.0001, and for RBC^CD24−^ Poisson regression chi-square for test article concentration effect =83.82, *p* < 0.0001). Moreover, a higher proportion of mutant cells were consistently observed in the RET cohort compared to the RBC cohort; however, in both cell populations, each dose group was significantly elevated compared to control (*p* < 0.05). One mouse, treated with a single i.p. dose of ENU (45 mg/kg body weight) was used as a positive control, and it also showed an increased frequency of RET^CD24−^ and RBC^CD24−^. There also appeared to be a slight increase in % RETs with increasing BaP dose. This effect has also been observed in BaP-treated rats [Bhalli et al.,2011b], although it should be noted that BaP itself endows RBCs with a modest amount of green fluorescence, and this may have interfered with flow cytometric quantification of % RETs.

#### lacZ Mutation Evaluation

**Fig. 3:**
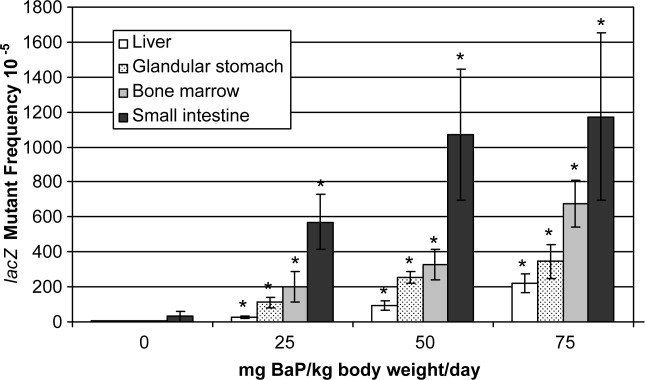
Frequency of *lacZ* mutants observed in liver, bone marrow, glandular stomach and small intestine of BaP-exposed Muta™ Mouse. Bars represent the mean of measurement from five separate animals and error bars represent the standard error of the mean. (* indicates *p* < 0.05 compared to control).

MF for the *lacZ* transgene was evaluated in four tissues (liver, bone marrow, glandular stomach, and small intestine) 72 hr following the last dose. Figure 3 summarizes the mutagenic response at the *lacZ* transgene. A dose-dependent increase in BaP-induced *lacZ* MF was observed in all four tissues (Poisson regression chi-square for test article concentration effect = 149.56 for bone marrow, 237.95 for liver, 156.95 for small intestine and 179.09 for glandular stomach, *p* < 0.0001 for all tissues.) Small intestine consistently yielded the highest frequency of *lacZ* mutants, followed by bone marrow, glandular stomach, and liver. In all tissues, all doses of BaP yielded a frequency of *lacZ* mutants that was significantly elevated compared to control (*p* < 0.005). Spontaneous MF (×10^−5^) for each of the tissues are as follows (standard error in brackets): liver: 6.31 (1.30), bone marrow: 3.97 (1.39) and glandular stomach: 5.95 (2.71), small intestine: 32.77 (30.36).

### Micronucleus Formation

**Fig. 4:**
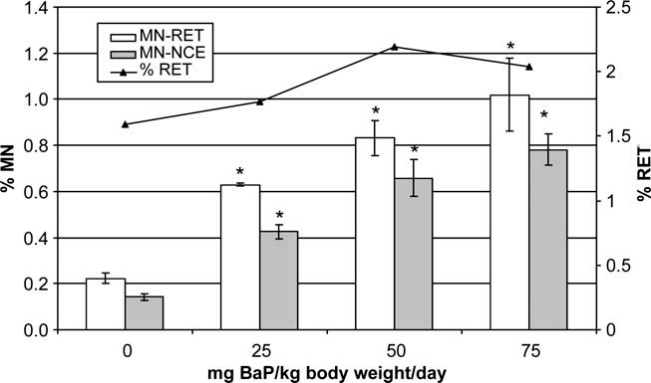
Frequency of micronucleated reticulocytes (% MN-RET) and micronucleated normochromatic erythrocytes (% MN-NCE) in BaP-exposed Muta™ Mouse. Bars represent the mean of measurement from five separate animals and error bars represent the standard error of the mean. (^*^denotes *p* < 0.005 compared with control).

We also measured micronucleus frequency in blood from BaP-exposed animals collected 48 hr following the last treatment. Figure 4 summarizes these results. A dose-dependent increase in % MN-RETs and % MN-NCE was observed (for RETs, Poisson regression chi-square for test article concentration effect = 46.77, *p* < 0.0001, and for NCEs, Poisson regression chi-square for test article concentration effect = 112.53, *p* < 0.0001). In both cell populations, each dose group was significantly elevated compared to control (*p* < 0.05). A dose-dependent increase in % RET was also observed confirming target tissue exposure.

### Comparison of Responses across Endpoints

**Fig. 5:**
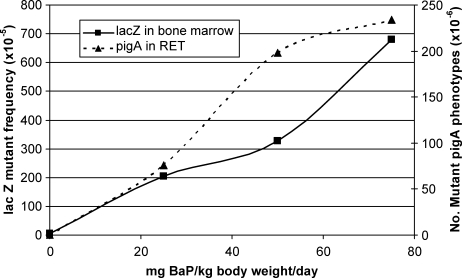
Dose-response relationship for BaP-induced mutants at the *lacZ* transgene in bone marrow and the frequency of mutant *Pig-a* phenotypes in reticulocytes (RETs). Note the difference in scale between the two *y*-axes.

An important component of the validation of the *Pig-a* assay is the comparison of the responsiveness of the mutant *Pig-a* phenotype endpoint to that of a well-established in vivo gene mutation assay. In this study, we compared the induction of BaP-induced mutant *Pig-a* phenotypes (as RET^CD24−^) to the induction of *lacZ* mutants in the bone marrow, in the same animals. As depicted in Figure 5 the dose-response relationships observed for *Pig-a* (i.e., RET^CD24−^) and *lacZ* MF in bone marrow, are similar. Linear regression analysis of *lacZ* MF versus the frequency of RET^CD24−^ cells at matched BaP doses revealed a strong correlation (*r*^2^ = 0.94, *p* < 0.05). Interestingly, the maximum *lacZ* MF in bone marrow was approximately 25-fold higher than the frequency of phenotypic mutants observed for *Pig-a* in RETs.

The BaP doubling dose (i.e., the dose of BaP required to elicit a doubling in the spontaneous frequency) for each of the endpoints measured in this study was calculated to permit a quantitative comparison of response induction across endpoints (Table I). Unlike other response metrics (e.g., mutation frequency), doubling dose permits comparison across endpoints and loci, regardless of the response unit. The BaP doubling doses for *lacZ* MF in all four tissues appear to be correlated with tissue-specific cell turnover rates, with relatively quiescent tissues such a liver showing higher doubling doses. Only the doubling doses calculated for liver and bone marrow are significantly different from each other (*p* < 0.05). Moreover, the doubling dose for RET^CD24−^ and RBC^CD24−^ are not significantly different from the *lacZ* doubling dose in bone marrow. However, the doubling dose for micronucleus formation is an order of magnitude higher than that calculated for the gene mutation endpoints (*lacZ* or *Pig-a*), and significantly different (*p* < 0.05).

**Table I:** BaP Doubling Doses Calculated For *lacZ* Mutant Frequency, Mutant *Pig-a* Phenotypes and Micronucleus Frequency

Endpoint	Tissue	BaP doubling dose (mg BaP/kg body weight/day)	Standard deviation
*lacZ* mutant frequency	Bone marrow	0.458	0.249
	Small intestine	2.12	59.7
	Glandular stomach	1.28	1.57
	Liver	2.17	0.581
Mutant *Pig-a* phenotypes	RET	0.0856^a^	0.106
	RBC	0.346	0.272
Micronucleus frequency	RET	21.7	0.29
	RBC	16.8	0.0882

a Calculated using the spontaneous frequency of RET^CD24-^ from subsequent Muta™ Mouse analyses performed using the newer immunomagnetic scoring procedure (0.28 ± 0.23 × 10^−6^). Doubling dose for this endpoint could not be calculated using the method employed here since no RET^CD24−^ were detected in unexposed animals.

Mutagenic efficiency, indicative of the rate of conversion of DNA adducts into mutations (expressed as number of mutants per DNA adduct in 10^8^ nucleotides at a given dose), was calculated for *lacZ* mutants in bone marrow and mutant *Pig-a* phenotypes in RETs. The mutagenic efficiency of *lacZ* in bone marrow was found to be 25-fold higher than that for RET^CD24−^ (Table II). However, it is important to note that since it was not possible to measure DNA adducts directly in the RET population, we have estimated the mutagenic efficiency at the *Pig-a* locus based on the level of DNA adducts in the entire bone marrow. Accordingly, the mutagenic efficiency for *Pig-a* could not be adjusted for the proportion of bone marrow DNA adducts in RET or RBC progenitor cells.

**Table II:** Mutagenic Efficiency at the *lacZ* and *Pig-a* Loci

Endpoint	Mutagenic efficiency (mutants per DNA adduct in 10^8^ nucleotides)
*lacZ* mutants in bone marrow	137 × 10^−6^
mutant *Pig-a* phenotypes in RET	5.47 × 10^−6^

## DISCUSSION

In this study we have carried out a subchronic, 28-day BaP exposure in Muta™Mouse in an effort to compare the response of mutant *Pig-a* phenotype formation with that of mutation formation in the well-studied *lacZ* transgene. We have shown that these mutational endpoints, along with measurements of internal dose (i.e., DNA adducts) and chromosomal damage (i.e., micronucleus formation), can be effectively integrated into a 28-day repeat dosing study, thus reducing the number of animals required in subchronic genotoxicity studies.

The presence of DNA adducts in these animals shows that BaP was converted to its active metabolite (i.e., BPDE) and delivered to target tissues. The relative levels of adducts observed across tissues is not surprising (liver > glandular stomach > small intestine > bone marrow), given the route of exposure (i.e., oral gavage) and pharmacokinetic factors at play. In this study, the primary site of contact for BaP is the stomach, followed by the small intestine. BaP is then carried to the liver, the primary site of systemic metabolism, via hepatic portal circulation. BaP and its metabolites must enter systemic circulation in order to reach the bone marrow. Accordingly, the levels of DNA adducts are highest in glandular stomach and liver, the sites of contact and primary site of metabolism, respectively, and lowest in the bone marrow and small intestine (Fig. 1). Differences in the levels of metabolizing enzymes (e.g., P450) are also known to vary between tissues, and may account for some of the differences observed in DNA adduct levels.

Dose-dependent increases in both mutant *Pig-a* phenotypes (in RETs and RBCs) and *lacZ* mutants (in bone marrow, liver, glandular stomach and small intestine) were observed. The rate of conversion of DNA adducts into mutations, estimated using the ratio of mutant frequency to adduct frequency, and denoted as mutagenic efficiency, was calculated for both of these mutation targets in order to compare susceptibility of the two loci. We estimate that the efficiency of converting BaP-DNA adducts in the bone marrow into *lacZ* mutants is 25-fold higher than for mutant *Pig-a* phenotypes in RETs. Similarly, the magnitude of the *lacZ* response in bone marrow is approximately 25-fold higher than the *Pig-a* response in RETs, suggesting that the rate of conversion of adducts is slower for the *Pig-a* endpoint compared with the *lacZ* endpoint. In contrast, the BaP doubling dose for the *lacZ* mutation endpoint in bone marrow is not significantly different from the the *Pig-a* endpoint (in both RETs and RBCs) (Table I). Taken together, these results suggest that, although the rate of conversion of adducts into mutations may be different for *lacZ* and *Pig-a*, the kinetics of mutation accumulation for both mutation targets is the same once a mutation is fixed. The similarity in doubling dose is not unexpected given that both assays are designed to measure gene mutation events, and therefore involve the same mechanism of action. Interestingly, the BaP doubling doses calculated here for the *lacZ* endpoint also seem to increase with decreasing mitotic index of the tissue. This is also expected, since replication is required for mutation fixation and tissues such as liver have previously been highlighted for low cell turnover [Mirsalis et al.,^1989^].

Assuming that both the *lacZ* and *Pig-a* genes are neutral (i.e., cells with *lacZ* or *Pig-a* muations are not selected for or against in situ) [Cosentino and Heddle,^1996^; Keller et al.,^1999^; Rosti,^2002^; Lambert et al.,^2005^], it might be predicted that their mutagenic efficiencies should be the same; however, this does not appear to be the case. Differences in the mutagenic efficiency and the (related) magnitude of response between the two mutation endpoints may be explained by several factors. First and foremost, the discrepancy reflects the fact that the population of bone marrow cells evaluated for *lacZ* mutants differs from the population of cells evaluated for *Pig-a* mutations. Since we could not measure adducts directly in blood cells, for the purposes of the efficiency calculation, we have assumed that all bone marrow cells are capable of becoming RETs and RBCs. However, it is well known that bone marrow is composed of many different stem cell populations, only a fraction of which are hematopoietic stem cells (HSC), and, only a fraction of these HSC are committed to the erythrocyte population. The remainder of the HSCs are either destined to become lymphoid progenitor cells (i.e., lymphocytes or natural killer cells, etc.), or other myeloid-based blood cells (e.g., mast cells, macrophages, thrombocytes). Although measurement of the proportions of cell types in the bone marrow that are committed to becoming RETs would prove challenging, this information would permit a better estimate of mutagenic efficiency for the *Pig-a* endpoint. It is also important to note that DNA adducts were measured in the whole genome, whereas *lacZ* mutants and mutant *Pig-a* phenotypes are only measured at a specific locus and there may be some variability in adduct levels between the measured locus compared to the whole genome.

It is important to note that the BaP doubling dose for mutant *Pig-a* phenotypes in RETs (Table I) was calculated using the spontaneous frequency of RET^CD24−^ from other analyses performed using the newer high throughput scoring procedure [data not shown, method adapted for mice from Dertinger et al. (2011)]. The BaP doubling dose for the *Pig-a* endpoint in RET could not be calculated using the scoring method used for this study since no RET^CD24−^ were detected in unexposed control animals in this study, and a measurable level of background mutant *Pig-a* phenotypes is required to calculate doubling dose (see Eq. 1). This newer scoring method employs cell separation by paramagnetic particles to dramatically increase the number of cells scored, thus resulting in more accurate measurements of spontaneous frequency of RET^CD24−^.

Although the doubling dose values did not reveal any significant difference in the sensitivity of the two loci to BaP, there are marked differences in the absolute values of the mutant and phenotypic frequencies for the *lacZ* and *Pig-a* loci, respectively. The high MF at the *lacZ* locus relative to the *Pig-a* locus is analogous to the findings of Skopek et. al. [1996] in their comparison of *hprt* and *lacI*. The background mutation frequency in the *lacI* transgene of BigBlue™ rat was found to be approximately an order of magnitude higher than that of *hprt* [Skopek et al.,^1996^]. Here, we note a difference between the *lacZ* MF and the mutant *Pig-a* phenotype frequency of two orders of magnitude for spontaneous, and greater than one order of magnitude for induced frequency (mean = 24).

It should also be noted that older animals, such as those used in this study, are known to have a higher frequency of spontaneous mutations in *lacZ*, and other reporter genes; however, we have no reason to expect that the sensitivity of the mutation assays will be impaired. Moreover, the OECD guideline for TGR assays (no. 488) was not available at the time that the animal work was carried out, and no guidelines regarding animal age were available. Future work of this nature should adhere to the OECD guideline [OECD,^2011^].

Another important consideration is the fact that the *Pig-a* gene is subject to transcription-coupled repair in the mouse, while the *lacZ* transgene is not transcribed [Lambert et al.,^2005^]. This difference would presumably contribute to a higher frequency of mutations at the *lacZ* locus. There is also the possibility that a higher frequency of silent mutations (i.e., mutations that have no effect on protein function) occur in either one of the *lacZ* or *Pig-a* loci, and this contributes to inaccuracy of the measured mutant frequency. Other unknown factors, such as the sequence context and genomic location, may also contribute to the higher mutagenic efficiency observed for *lacZ*. For example, Lichtenauer-Kaligis et al. (1993) have shown that integration of *hprt* cDNA into different locations in the human TK6 genome results in differences in mutability.

We have also integrated measurements of chromosome damage into this 28-day repeat dose study by measuring the micronucleus frequency in RETs and NCEs. These measurements were made 48 hr following the last exposure in accordance with OECD guideline 474 [OECD,^1997^]. Integration was quite simple since only a small blood sample is required for the analysis, and it can be obtained from a living animal (e.g., by saphenous or submandibular vein bleed). The same animals can then be used for analysis of mutations and DNA adducts at three days following the last treatment, thereby reducing the total number of animals required for the study. Integration of multiple endpoints into subchronic repeat dose studies has been highlighted as a way to reduce animal numbers in genetic toxicology studies [Pfuhler et al.,^2009^]. A recent study showed that the micronucleus assay, comet assay, and *Pig-a* assay can be integrated into a 28-day repeat dose study in rats [Shutsky et al.,^2010^], and measurements of micronucleus frequency have been successfully carried out alongside the *Pig-a* assay in a 28-day subchronic rat study [Dertinger et al.,^2010^].

In contrast to the gene mutation endpoints, the dose-response kinetics of micronucleus formation were quite different; the BaP doubling dose calculated for micronucleus formation in RETs is approximately 10-fold higher than those for the *Pig-a* and *lacZ* mutation endpoints. This observation is consistent with the current understanding of the different mechanisms of micronucleus versus mutation formation. Neutral gene mutations have no selective advantage or disadvantage, and hence, cells containing new neutral mutations can accumulate over time during continuous treatment of the exposed tissue. In contrast, micronuclei measured in RETs are formed during mitosis and cannot accumulate with multiple cell divisions since the MN-RET (i.e., polychromatic erythrocytes) are expected to mature and enter the population of red blood cells (i.e., NCEs).

In this study, we used the standard OECD TGR assay testing regime of 28 + 3 days (i.e., 28 day exposure period + 3 day sampling time) since this is the minimum time necessary for detection of mutations in the *lacZ* gene [Thybaud et al.,^2003^; OECD,^2011^]. We observed significant dose-dependent increases in BaP-induced *Pig-a* mutant RET^CD24−^ and RBC^CD24−^ phenotypes and *lacZ* mutants (Figs. 2 and 3, respectively). At the three day sampling time, however, a higher proportion of mutant *Pig-a* phenotypes is observed in the RET population (versus RBC). This has been shown previously, and is due to the time required for the turnover of RETs into RBCs. In mice, the maximum RET response is reached after 2weeks; whereas, the maximum RBC response is reached only after four weeks [Bhalli et al.,2011a]. The relatively higher response observed in RETs may also be due to the reduced lifespan of GPI-deficient RBC due to their susceptibility to complement-mediated lysis [Tremml et al.,^1999^]. Likely for these same reasons, we also observed a higher mutagenic efficiency in RETs versus RBCs (Table II). Although the sampling time of 3 days post-exposure may not be optimal for the maximum *Pig*-*a* response, it is sufficient to observe dose-related effects and, importantly, is compatible with integration into a TGR assay.

It is also possible that the 28+3 sampling regime is not ideal for measuring DNA adducts; however, it is known that adducts formed by BaP are relatively persistent following acute dosing [Stowers and Anderson,^1985^]. We are not aware of previously published information on the kinetics of formation and elimination of BPDE DNA adducts following a 28-day subchronic repeat dose study in the mouse, however, studies by Poirier et al. conducted in rats may help shed some light on murine DNA adduct kinetics. In Poirier et al. [1982], Wistar-Furth rats were fed 2-acetylaminofluorene for 28-days and the kinetics of hepatic DNA adduct formation and elimination were measured. Adduct levels reached a plateau at approximately two weeks, and once treatment stopped, adduct levels began to decrease. However, at three-days post-treatment greater than 90% of adducts still remained [Poirier et al.,^1982^]. Based on this information, and the fact that the measured levels of BaP-DNA adducts showed dose-dependency and strong correlation with *lacZ* mutant frequency, it is safe to say that the BaP-DNA adduct levels at 28 days were at steady state, and the adduct levels measured at 72-hr post-exposure were a sufficiently accurate representation of BaP internal dose. Nevertheless, it should be acknowledged that the level of BPDE adducts observed in a tissue at any given time depends on the balance between the metabolic conversion of BaP to BPDE (via phase I metabolism), detoxification (via phase II metabolism), the rate of adduct repair, and the cell turnover rate. Therefore, although is seems plausible that the DNA adduct levels on day 28 were slightly higher than those at three days post-treatment, the sampling time required for evaluation of *lacZ* mutant frequency did not permit assessment of adduct frequency immediately following administration of the final dose. It may be prudent to investigate the kinetics of adduct loss following subchronic oral BaP exposures in order to validate the efficacy of simultaneous measurements of numerous endpoints in the same animals.

A major hurdle in the validation of the *Pig-a* assay has been confirmation of the mutational basis for the observed phenotypes (i.e., GPI-anchor deficiency); however, sequencing of *Pig-a* mutant blood cells is not possible since RBCs lack nuclear DNA. Nevertheless, *Pig-a* mutations have been confirmed in GPI-anchor deficient rat splenic T-cells [Miura et al.,^2008^; Miura et al.,^2011^], and in patients with the GPI-anchor deficiency disorder, paroxysmal nocturnal hemoglobinurea (PNH) [Nishimura et al.,^1999^]. Compelling evidence for the mutational origin of GPI-anchor deficient phenotypes in the *Pig-a* assay was most recently presented by Kimoto et al (2011). In that study, cDNA from CD24-negative murine bone marrow cells was sequenced, and it was shown that mutations in *Pig-a* are present when CD24 is lacking [Kimoto et al.,^2011^]. Evidence of a similar doubling dose for the *Pig-a* locus and the more commonly employed *lacZ* locus provides further evidence for the mutational origin of GPI-anchor deficiencies observed in RBCs and RETs in the *Pig-a* assay, since the result implies a similar mechanism of action.

In summary, we have demonstrated that multiple endpoints can be incorporated into a 28-day subchronic mouse toxicity study, and the results obtained support the validation of the *Pig-a* assay by showing that the assay yields comparable responses (i.e., doubling dose) to the commonly used and validated transgenic rodent mutation assay, thus providing further support regarding its utility as an in vivo somatic gene mutation assay. Although differences in mutagenic efficiency were observed between the two gene targets, we hypothesize that the differences are likely due to difficulties in assessing adduct frequency in the target cell populations, and differences in repair capacity across loci, rather than differences in the rate of conversion of adducts into mutations.

Future studies should aim to further investigate the responsiveness of GPI-anchor deficient cells in the *Pig-a* assay relative to more established in vivo genetic toxicity endpoints such as *lacZ*, including investigations of the low dose region of the dose-response function. Moreover, 28-day repeat dose studies that test additional classes of compounds with different modes of action, as well as time-course studies to examine the persistence of mutant *Pig-a* phenotypes will provide further insight into the utility of the assay, and will be important steps in the validation of the *Pig-a* endpoint as a useful and effective in vivo somatic gene mutation assay.
